# Rare Earths as Authenticity Markers for the Discrimination of Greek and Turkish Pistachios Using Elemental Metabolomics and Chemometrics

**DOI:** 10.3390/foods10020349

**Published:** 2021-02-07

**Authors:** Natasa P. Kalogiouri, Natalia Manousi, Dimitris Klaoudatos, Thomas Spanos, Vilson Topi, George A. Zachariadis

**Affiliations:** 1 Laboratory of Analytical Chemistry, Department of Chemistry, Aristotle University of Thessaloniki, 54124 Thessaloniki, Greece; nmanousi@chem.auth.gr (N.M.); zacharia@chem.auth.gr (G.A.Z.); 2 Laboratory of Oceanography, Department of Ichthyology and Aquatic Environment, School of Agricultural Sciences, University of Thessaly, 38446 Volos, Greece; dklaoud@uth.gr; 3 Laboratory of Instrumental Analysis, Department of Chemistry, International Hellenic University, 65404 Kavala, Greece; tspanos@chem.ihu.gr; 4 Department of Forest and Natural Environment Sciences, International Hellenic University, 1st km Drama-Microchoriou, 66100 Drama, Greece; vilson.topi@gmail.com

**Keywords:** authenticity, nuts, PDO, Greek pistachios, rare earths, ICP-MS, chemometrics

## Abstract

Pistachios are a nutritionally beneficial food source widely consumed all over the world. Pistachios exhibit high content of antioxidants, vitamins and other beneficial micronutrients, including nutrient elements and rare earth elements (REEs). Considering that the concentration of REEs depends on the climate and soil characteristics that vary among different geographical regions, REEs could constitute markers responsible for the geographical discrimination of this nut type. In this study, Greek pistachios with a protected designation of origin (PDO) label from Aegina Island and Fthiotida and Turkish pistachios from Adana were analyzed with inductively coupled plasma mass spectrometry (ICP-MS) to assess their REE profile. La, Ce, Pr, Nd, Sm, Eu, Gd, Tb, Dy, Ho, Er, Tm and Yb were determined and quantified. The quantification results were further analyzed using the main effect plot, permutational analysis of variance (PERMANOVA), nonmetric multidimensional scaling (nMDS), principal component analysis (PCA) and hierarchical clustering (HCA) to investigate the similarities between the pistachios. A decision tree (DT) was developed for the classification of pistachios according to their geographical origin proving to be a promising and reliable tool for verifying the authenticity of food products on the basis of their REE profile.

## 1. Introduction

Pistachios, *Pistachia vera*, are considered as one of the most economically and nutritionally available food sources and are widely consumed all over the world, both for their taste and health benefits [1]. They are rich in antioxidants, including phenolic compounds, vitamins, carbohydrates, minerals and beneficial nutrients [2,3]. The mineral composition of pistachios, like other nuts and agricultural products, is influenced by a variety of factors, including the climate and soil characteristics including the organic matter content, the pH value and the clay mineralogy. Since these factors vary among different regions, the elemental composition of nuts can be dependent on their cultivation place [2,4].

Rare earth elements (REEs) are a set of seventeen transition metals including scandium (Sc), with an atomic number of 21; yttrium (Y), with an atomic number of 39; and the 15 lanthanides, with atomic numbers ranging from 57 to 71, as defined by the International Union of Pure and Applied Chemistry (IUPAC). Lanthanides consist of lanthanum (La), cerium (Ce), praseodymium (Pr), neodymium (Nd), promethium (Pm), samarium (Sm), europium (Eu), gadolinium (Gd), terbium (Tb), dysprosium (Dy), holmium (Ho), erbium (Er), thulium (Tm), ytterbium (Yb) and lutetium (Lu) [5,6]. 

In the past years, REEs had multiple applications in the field of agriculture, including their use as fertilizers, growth promoters and feed additives. As a result, during recent years, the concentration of REEs in samples of environmental origin has increased, resulting in increase in their occurrence in biological samples by food chain accumulation [7,8,9]. REEs can be released from environmental samples such as soil and water and can enter the human body via a wide range of exposure pathways and especially food ingestion [10]. The long-term exposure to REEs may lead to accumulation in the blood, bones and brain after entering the human body [10,11]. Moreover, the intake of REEs has been related with DNA stability impairment or DNA damage [8,12]. As a result, the determination of REEs in edible products is of high importance because of their increasing occurrence and their toxicity [13]. 

REEs have also been proposed for use as chemical markers for origin discrimination of various natural products with the application of a broad range of statistical and pattern recognition methods [14]. The importance of the statistical analysis of the analytical data and the development of robust chemometric models in food authenticity studies has already been reviewed in detail [15,16,17]. The vast majority of the works published in the literature discuss the fingerprinting of foods based on their bioactive profile [18,19] and the determination of markers that belong to the class of polyphenols [16,20,21]. Limited works, however, have been implemented for the determination of REEs and the investigation of authenticity based on the REE profile of foods [22,23,24,25] using inductively coupled plasma mass spectrometry (ICP-MS).

The aim of the study was to assess the REE profile of pistachios originating from Greece—PDO-labelled “Fthiotida Pistachio” and “Aegina Pistachio”—and from Adana, Turkey, with ICP-MS, considering that the origin of plant-based products is believed to have a direct impact on its REE profile. To the best of our knowledge, this is the first study that reports the determination of REEs, namely La, Ce, Pr, Nd, Sm, Eu, Gd, Tb, Dy, Ho, Er, Tm and Yb, in pistachios of different origin. The determined REEs were further analyzed using the main effect plot, permutational analysis of variance (PERMANOVA), nonmetric multidimensional scaling (nMDS), principal component analysis (PCA) and hierarchical clustering (HCA) to investigate the similarities between the pistachios, and a decision tree (DT) was developed for their classification.

## 2. Materials and Methods

### 2.1. Materials and Reagents

For the acidic digestion, supra pure nitric acid 65% was supplied Merck (Darmstadt, Germany). Double deionized water was prepared by passing through two ion exchange columns.

Stock standard solutions for the REEs (La, Ce, Pr, Nd, Sm, Eu, Gd, Tb, Dy, Ho, Er, Tm and Yb), at a concentration of 1.000 mg/L, were prepared by dissolving appropriate amounts of their nitrate salts (Pfaltz & Bauer Inc., Waterbury, CT, USA) in 2% *v/v* supra pure HNO_3_. Subsequently, working standard solutions were prepared by appropriate serial dilutions of their stock solutions into 2% *v/v* suprapure HNO_3_.

### 2.2. Sample Collection

**Figure 1:**
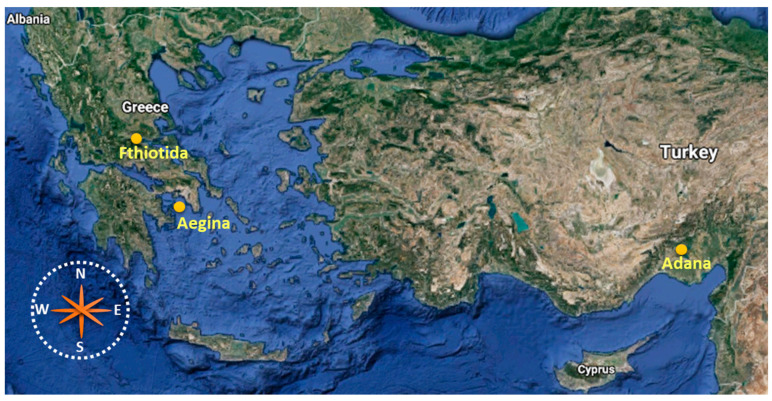
Geographical origins of Greek and Turkish pistachios originating from Aegina, Fthiotida and Adana (Source: https://www.google.com/maps, modified in Microsoft Office 2019 (Microsoft, WA, USA)).

Seventeen pistachios samples from conventional cultivars were collected from Greece (8 from Fthiotida and 9 from Aigina), and 11 samples were acquired from Adana, Turkey, during the crop years οf September 2019 and 2020. The pistachios were harvested manually. All samples were preserved in a dark and cold room before analysis. The geographical origins of the collected samples are presented in Figure 1. 

### 2.3. Sample Preparation

The concentration of REEs was determined in pistachios from three different geographical origins (Aegina, Fthiotida and Adana). First, the nut samples were oven-dried at 50 °C for 24 h. For digestion of the samples, about 500 mg was weighed, ground and placed into polytetrafluoroethylene (PTFE) vessels for acid digestion. Subsequently, 5 mL of nitric acid was added into the vessel and the samples were subjected to autoclave digestion for 60 min at 150 °C. After digestion, the vessels were cooled to room temperature and opened. Accordingly, the obtained clear solution was transferred into a 25 mL volumetric flask and the volume was made up to the mark with double-deionized water. 

### 2.4. ICP-MS Analysis 

For the determination of REEs in nut samples, an Agilent HP-7700x Series ICP-MS (Agilent, Santa Clara, CA, USA) instrument was employed. The working parameters of the ICP-MS are as follows: 1.55 kW reflect power; 15.0 L/min plasma gas flow rate; 0.95 L/min carrier gas flow rate; 0.75 L/min nebulizer flow rate; concentric nebulizer, Scott spray chamber, continuous acquisition mode, 27 MHz RF generator; and 3 replicates.

Calibration curves were constructed from rare earth standard mixtures after dilution of the stock solutions over the range 5–1000 ppt. Limits of detection (LOD) and limits of quantification (LOQs) were calculated on signal-to-noise ratios of 3 and 10, respectively. The quantified isotopes were ^139^La, ^140^Ce, ^141^Pr, ^146^Nd, ^147^Sm, ^153^Eu, ^158^Gd, ^159^Tb, ^163^Dy, ^165^Ho, ^166^Er, ^169^Tm and ^172^Yb. All samples were analyzed in triplicate. 

The digestion of nut samples was performed into Teflon^®^ vessels (DuPont, DE, USA) that were placed in a steel autoclave and heated in a six-position aluminum block (Berghof, BTR,941, Eningen, Germany). The maximum working volume of the digestion system was 50 mL, while its maximum temperature was 180 °C. Deionized water was generated by a Milli-Q plus purification system (Millipore, Bedford, MA, USA).

The sample preparation apparatus, including glassware, digestion vessels, storage bottles, etc., were soaked in freshly prepared 10% (*v*/*v*) nitric acid for at least 48 h and washed several times with double deionized water prior to the experimental procedure, in order to avoid contamination. Moreover, crucibles were immersed in 20% (*v*/*v*) HCl for a period of two days and rinsed with double deionized water several times prior to their use for the next experiment.

### 2.5. Chemometrics

Chemometric analyses were performed using Minitab 19 software (Minitab, PA, USA) at an alpha level of 0.05. The main effect plot was used to examine the differences between the variables and the groups. All multivariate analysis was performed with the PRIMER package (PRIMER-e, Auckland, New Zealand). N-MDS was used as a means of visualizing the level of similarity between samples, based on the of Bray–Curtis similarity index using the unweighted pair group method with arithmetic mean (UPGMA) [26]. To normalize data and to avoid skewness, a log transformation was applied on the data prior to calculating similarities. PERMANOVA, a nonparametric multivariate statistical test, was used to assess the difference between groups [27]. PCA was used to analyze the interrelationships among variables. HCA was based on the Bray–Curtis similarity index. A DT was developed in the Matlab R2020a program (Mathworks) to classify the samples according to their geographical origins.

## 3. Results and Discussion

### 3.1. Chemical Analysis

The analytical parameters of the ICP-MS methodology are presented in Table 1. LODs and LOQs were relatively low over the range 0.016 (Ho)–0.060 (Pr) ng/kg and 0.030 (Ce)–0.533 (Sm) ng/kg, respectively. The calibration curves were linear over the range 5–1000 ppt for La, Ce, Pr, Gd, Tb, Dy and Ho and over the range 5–500 ppt for Nd, Sm, Eu, Er, Tm and Yb.

All samples were analyzed in triplicate, and the quantification results expressed as ng/g (±SD, *n* = 3) are presented in the Supplementary Materials, in Table S1. Among the examined rare earth elements, Sm was the most abundant. The concentration of Nd ranged between 1224–1680 ng/kg for pistachios from Fthiotida and between 148–1985 ng/kg for pistachios from Aegina, while lower concentration values (3.85–9.85 ng/kg) were observed for pistachios from Adana. La and Ce were determined in relatively high concentrations in pistachio samples, regarding the concentration levels of all the examined analytes. The concentration for La ranged between 215–4511 ng/kg for pistachios from Fthiotida, between 155–5161 ng/kg for pistachios from Aegina and between 5.76–63.6 ng/kg for pistachios from Adana. The concentrations of Ce ranged between 363–548 ng/kg for pistachios from Fthiotida, between 291–483 ng/kg for pistachios from Aegina and between 1.42–11.5 ng/kg for pistachios from Adana. Furthermore, the concentration of Eu ranged between 101–194 ng/kg for pistachios from Fthiotida, between 16.8–47.1 ng/kg for pistachios from Aegina and between 1.97–9.27 ng/kg for pistachios from Adana. The concentration of Ho ranged between 56.6–80.8 ng/kg for pistachios from Fthiotida, between 11.2–56.6 ng/kg for pistachios from Aegina and up to 5.15 ng/kg for pistachios from Adana. The concentration of Er ranged between 51.9–1214 ng/kg for pistachios from Fthiotida, between 13.3–34.9 ng/kg for pistachios from Aegina and between 0.18–2.11 ng/kg for pistachios from Adana. The concentration of Tm ranged between 25.6–190 ng/kg for pistachios from Fthiotida, between 5.60–11.0 ng/kg for pistachios from Aegina and between 1.49–6.45 ng/kg for those from Adana. The concentration of Yb ranged between 10.5–179 ng/kg for pistachios from Fthiotida, between 10.3–35.0 ng/kg for pistachios from Aegina and between 1.15–4.35 ng/kg for pistachios from Adana.

Among the examined REEs, the lowest concentration levels were observed for Pr, the concentration of which ranged between 7.64–9.98 ng/kg for pistachios from Fthiotida and between 2.96–9.80 ng/kg for pistachios from Aegina, while it was not detected in most pistachio samples from Adana. Regarding Dy, wide variability was observed in the pistachio samples. Specifically, the concentration of Dy ranged between 56.9–300 ng/kg for pistachios from Fthiotida and between 13.5–70.2 ng/kg for pistachios from Aegina and was not detected in most pistachio samples from Adana. This was also the case for Tb, which was not detected in any pistachio samples from Adana, while its concentration levels for pistachios from Fthiotida and Aegina ranged between 29.8–97.7 ng/kg and up to 14.82 ng/kg, respectively. Nd was detected over the range 1224–1543 ng/kg in Fthiotida pistachios, between 148–1985 ng/kg in Aegina pistachios and between 0.39–0.99 ng/kg in pistachios from Adana, respectively. Finally, Gd was not detected in any samples originating from Adana and was detected at from 6.55 to 14.8 ng/kg in Aegina pistachios and over the range 29.8–82.4 ng/kg in Fthiotida pistachios.

The results of the concentration of the thirteen examined REEs were compared with results from the literature. In 2012, Guo et al. [28] performed a survey for the determination of rare earth elements in major foods in China and the average content of the examined elements ranged between 1.20–52.4 μg/kg, which is a lot higher than the concentration ranges reported in this study, probably due to the different geographical origin and environmental conditions. Moreover, the values for most rare earth elements were comparable with those reported in Greek fava samples [29] and cheeses [30].

### 3.2. Chemometric Analysis

#### 3.2.1. Main Effect Plot

**Figure 2:**
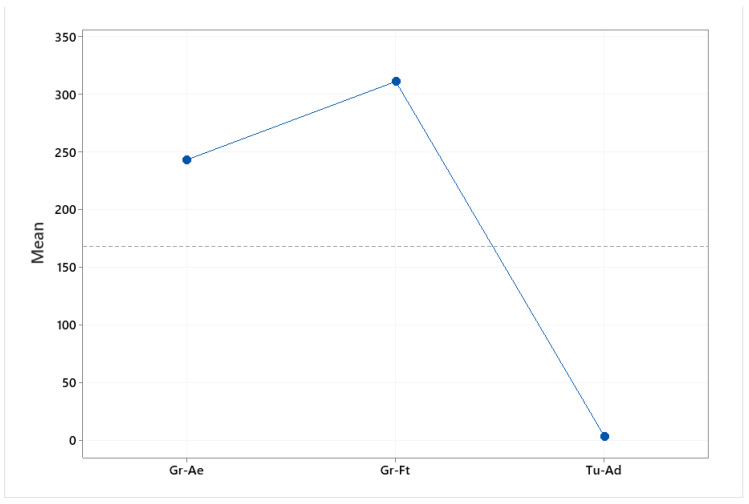
Main effects plot of the influence that each element exerts between the pistachios originating from Greece (Aegina, Fthiotida) and Turkey (Adana).

**Figure 3:**
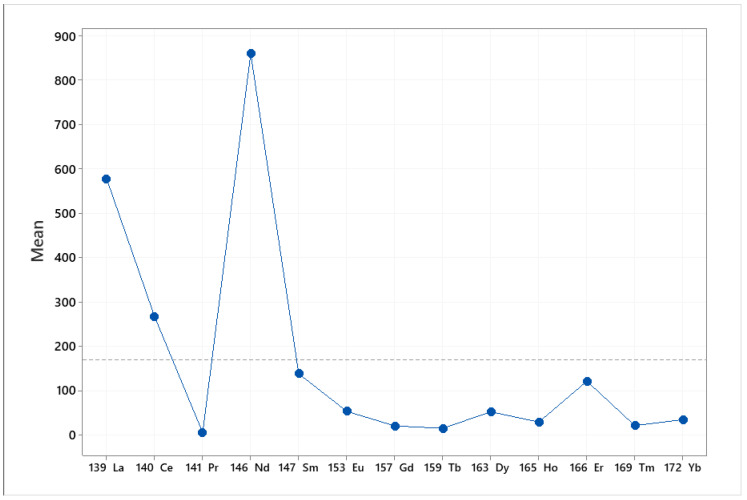
Main effects plot of the influence that geographical origin (Aegina, Fthiotida, Adana) exerts between samples.

The main effect plot is the graphical tool that presents the mean outcome for each independent variable’s value combining the effects of the rest of the valuables. The main effects plot was used to examine the differences between the rare earths’ values. There is a main effect when different levels of a factor affect the response differently. A main effects plot graphs the response mean for each factor level connected by a line. When the line is horizontal (parallel to the *x*-axis), then there is no main effect. Each level of the factor affects the response in the same way, and the response mean is the same across all factor levels. When the line is not horizontal, then there is a main effect. Different levels of the factor affect the response differently. The steeper the slope of the line, the greater the magnitude of the main effect. The influence that each factor (elements, origin) exerts on the differences observed between samples is indicated in Figure 2 and Figure 3. Specifically, Figure 2 indicates that Nd and La exert the greatest influence between the samples. Furthermore, Figure 2 indicates the great difference in the rare earths’ content between the pistachios originating from Aegina, Fthiotida and Adana. 

#### 3.2.2. Permutational Analysis of Variance

PERMANOVA is a nonparametric multivariate technique. It tests the simultaneous response of one or more variables to one or more factors on the basis of any distance measure with the use of permutation methods. Similar to ANOVA, PERMANOVA measures the sum-of-squares within and between groups and uses the F test to compare within-group to between-group variance. While ANOVA draws the significance of the result on consumption of normality, PERMANOVA compares the actual F test value to the one gained from random permutations of the objects between groups. It is applied to compare groups of objects and test the null hypothesis that the centroids and dispersion of the groups are equivalent for all groups. PERMANOVA was applied to compare the quantification results of the rare earths determined in all pistachios samples on the basis of their geographical origin. Data were log-transformed, unrestricted permutation of raw data was used and similarity was based on the Bray–Curtis similarity index. The results indicated that there is significant statistical difference between the PDO pistachios of Aegina and Fthiotida (*p* < 0.05) as well as between the pistachios from Adana, Turkey (*p* < 0.05). Table 2 presents all the statistical values for the pistachios of different origins, indicating significant difference between all sample groups.

#### 3.2.3. Nonmetric Multidimensional Scaling

**Figure 4:**
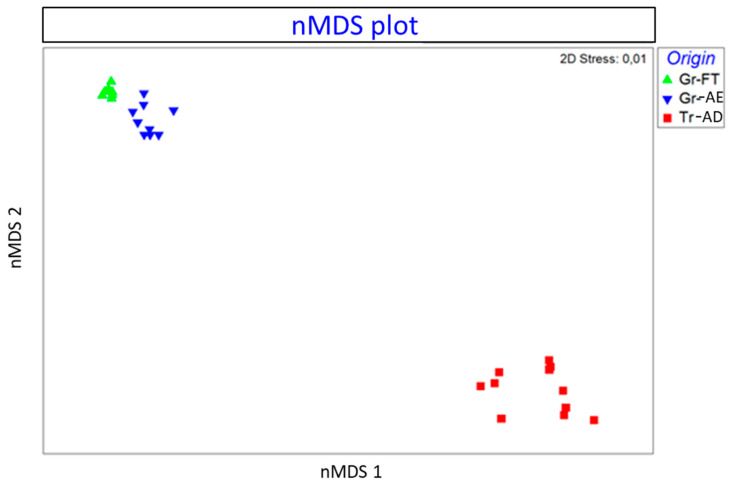
Schematic nonmetric multidimensional scaling (nMDS) plot presenting the similarity between pistachios: Gr-FT (Greece, Fthiotida), Tr-AD (Greece, Aegina) and Tr-AD (Turkey, Adana), based on the Bray–Curtis similarity index of log-transformed data. Closer points indicate higher similarity.

MDS represents the measurements of the distance between points of a low-dimensional space to depict similarity among pairs of samples in high-dimensional space [31]. The objective of nonmetric MDS is to arrange the input data objects into a low-dimensional space so that the new distances reflect the rank order of the data [32]. The method finds the optimal spatial dimension and position to reveal the true structural relationship between the objects. The degree of correspondence between the distances among points implied by an MDS map and the matrix input by the user is measured (inversely) by a stress function. The lower the stress value, the better the fit. Dimensions are similar to factors as they give the number of facets of the relationships between stimuli and may be plotted. Stress is standardized to bring values between 0 and 1. Dugard et al. [33] suggest that stress values around or below 0.1 are excellent and above 0.15 are unacceptable. The nMDS plot is presented in Figure 4. The points represent the samples. It is clearly shown that the three samples of pistachios from the same origins (Aegina, Fthiotida and Adana) are ordinated closer together, forming three groups according to the geographical origin. The Greek pistachios from the Fthiotida and Aegina groups are ordinated separately and at a greater distance from the pistachios originating from Adana. The stress value was calculated 0.01, reflecting that the ordination is adequate.

#### 3.2.4. Principal Component Analysis

**Figure 5:**
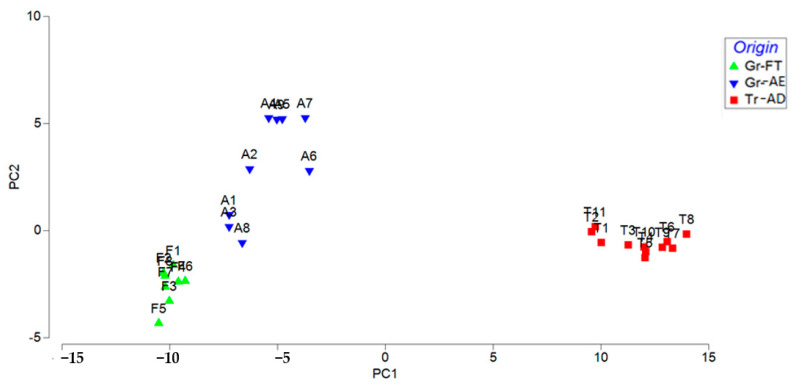
PCA plot showing pairwise correlations between principal components (PCs) and REE quantification results between the three groups of pistachios: Gr-FT (Greece, Fthiotida), Gr-AE (Greece, Aegina) and Tr-AD (Turkey, Adana).

PCA is a chemometric technique used for exploratory data analysis. PCA projects the data in a reduced hyperspace, which is defined by principal components (PCs). The PCs are linear combinations of the original variables and PC1 (first PC) exhibits the largest variance, PC2 the second-largest, and so on. PCA enhances data exploration and interpretation of multivariate datasets and shows the pattern of similarity of the observations by displaying them as points in the score plot. The score plot in Figure 5 presents the distribution of pistachios samples showing that the samples were classified in three groups on the basis of their geographical origin, with PC1 and PC2 explaining 92.4% of the total variation. The loading plot (Figure S1) and the PCA results (Table S2) can be found in the Supplementary Materials.

#### 3.2.5. Hierarchical Clustering

**Figure 6:**
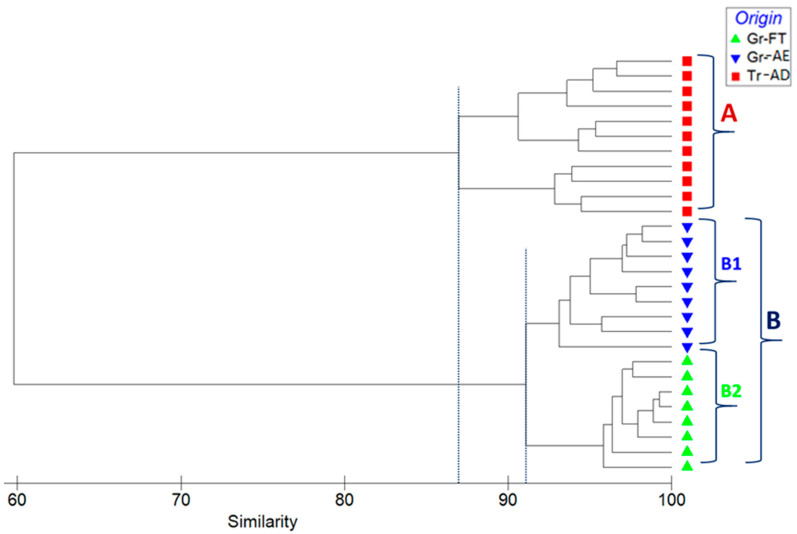
Dendrogram for hierarchical clustering of the elemental abundance of all samples, using group-average clustering of Bray–Curtis similarities based on Log(X + 1)-transformed abundances.

Cluster analysis is applied to divide a set of objects into a number of homogeneous groups or clusters where there is no a priori information about the group structure of the data [34]. Cluster analysis measures the distance between each pair of objects in terms of variables, grouping the objects that are close together. The dendrogram of hierarchical cluster analysis (Figure 6), based on Bray–Curtis similarity index, indicated the presence of two major groups of pistachios from Greece (group A) and Turkey (group B), with 87% mean similarity between the samples’ same class to the mean similarity between objects in the second class. Group B subdivides into two subgroups, PDO pistachios from Fthiotida (group B1) and PDO pistachios from Aegina (group B2), with 91% similarity.

#### 3.2.6. Decision Tree

**Figure 7:**
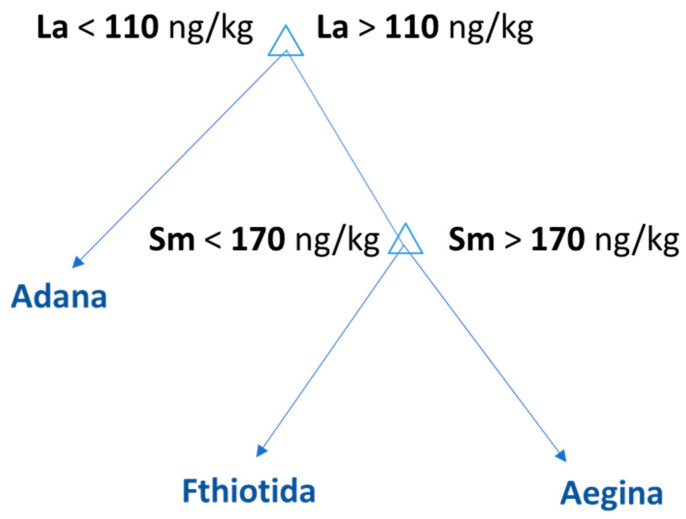
Decision tree model for the classification of the pistachios according to their geographical origin.

A supervised machine learning classification method was used to discover patterns in the quantitative data. The decision tree algorithm builds a model by splitting the data repeatedly into two distinct subsets according to the values of one explanatory variable. A single value is assigned to each subset for the predicted outcome. Each node splits, selecting the most significant variable that minimizes the model’s total error. The procedure is repeated within each new subset, splitting the data along one variable at a time, and each split can be represented as a node with two branches. The DT was developed in Matlab 2020b to classify the samples on the basis of their geographical origin. The ‘‘classregtree’’ function was used to determine the best split for each node (CART). The dataset was split into a training and a test set. Six pistachios originating from Fthiotida, seven from Aegina and seven from Adana were used to train the model, and two samples from each class were used as a test set. The developed DT suggested La and Sm as characteristic markers and successfully classified samples into three groups on the basis of their geographical origin. The developed regression tree was validated with receiver operating characteristics (ROC) plot for each class of pistachios with 1-specificity and zero error. Τhe ROC curves for its class are included in the Supplementary Materials (Figure S2). Figure 7 is the visual representation of the decision tree model developed for the classification of the pistachios according to their geographical origin. The DT reveals an interesting pattern with two splits. The top node classifies the samples as Turkish or Greek based on La concentration. According to the developed model, the pistachios with concentrations of La lower than 110 ng/kg originated from Adana. Following the right branch of the tree, the Greek samples with Sm concentrations above 170 ng/kg originate from Aegina and the pistachios with concentrations lower than 170 ng/kg originate from Fthiotida.

## 4. Conclusions

Pistachios from different geographical origins in Greece and Turkey were analyzed with ICP-MS for the determination of REEs. Twenty-eight samples originating from Aegina, Fthiotida and Adana were analyzed, investigating if the determined REEs could be used as markers for the classification of samples based on their geographical origin. La, Ce, Pr, Nd, Sm, Eu, Gd, Tb, Dy, Ho, Er, Tm and Yb were determined and quantified. The main effect plot indicated that Nd and La exert the greatest influence between the pistachio samples and that the REE composition differs among the pistachios originating from Aegina, Fthiotida and Adana. The nMDS plot showed that the pistachios originating from the same region (Aegina, Fthiotida or Adana) are ordinated closer together, forming three separate groups. PCA analysis confirmed the classification of the samples according to the geographical origin, with 92.4% explained variance. Finally, cluster analysis was performed and the dendrogram classified the pistachios into two main groups: Greek and Turkish with two subgroups of the latter—those originating from Fthiotida and Aegina. Finally, the DT classified selected two RREs, La and Sm, proposing a concentration threshold for their classification according to their geographical origin. This study contributed to the existing knowledge about the REE profile of pistachios and their potential use as authenticity factors. This is an exploratory study showing that the REE profile can be used for the accurate discrimination of nuts and could be expanded to different cases of food authenticity as well.

## Figures and Tables

**Table 1:** ICP-MS analytical parameters.

Element	Mass	Slope	Intercept	r^2^	LOD ng/kg	LOQ ng/kg	Linear Range
La	139	818	5826	0.992	0.023	0.077	5–1000
Ce	140	2875	7503	0.996	0.009	0.030	5–1000
Pr	141	1745	37568	0.996	0.060	0.200	5–1000
Nd	146	84	1713	0.995	0.129	0.430	5–500
Sm	147	70	1348	0.996	0.160	0.533	5–500
Eu	153	209	1919	0.993	0.027	0.090	5–500
Gd	157	204	3854	0.997	0.096	0.320	5–1000
Tb	159	708	12315	0.992	0.077	0.257	5–1000
Dy	163	72.6	1185	0.991	0.120	0.400	5–1000
Ho	165	226	1021	0.994	0.016	0.053	5–1000
Er	166	115	462	0.991	0.040	0.133	5–500
Tm	169	214	845	0.996	0.025	0.083	5–500
Yb	172	70.6	213	0.993	0.031	0.103	5–500

LOD: limit of detection, LOQ: limit of quantification.

**Table 2:** PERMANOVA analysis results for pistachios from Aegina (Gr-AE), Fthiotida (Gr-FT) and Adana (Tr-AD).

Groups	Test Statistic (t)	Probability (p)	Unique Permutations
Gr-FT, Gr-AE	5.0311	<0.05	970
Gr-FT, Tr-AD	15.078	<0.05	981
Gr-AE, Tr-AD	12.716	<0.05	992
Average similarity between/within groups			
	Gr-FT	Gr-AE	Tr-AD
Gr-FT	96.907		
Gr-AE	91.09	94.661	
Tr-AD	56.302	62.876	89.715

## Supplementary Materials

The following are available online at https://www.mdpi.com/2304-8158/10/2/349/s1, Table S1. REEs quantification results (ng/kg); Figure S1. PCA loading plot; Table S2. PCA Results; Figure S2. ROC curves for pistachios (a) originating from Adana; (b) Fthiotida; (c) Aegina.
