# Supplementary material for: Rare Earths as Authenticity Markers for the Discrimination of Greek and Turkish Pistachios Using Elemental Metabolomics and Chemometrics
Source: Foods. 2021 Feb 7;10(2):349. doi: 10.3390/foods10020349 (PMC7915317; doi:10.3390/foods10020349)
Supplement: Supplementary file 1 [file foods-10-00349-s001.pdf]

Supplementary Material

Rare earths as authenticity markers for the discrimination of Greek and Turkish pistachios using elemental metabolomics and chemometrics

Natasa P. Kalogiouri, Natalia Manousi, Dimitris Klaoudatos, Thomas Spanos, Vilson Topi and George E. Zachariadis

Table S1. REEs quantification results (ng/kg)

	Fthiotida			Aegina			Adana		
**REEs**	**min**	**max**	**median**	**min**	**max**	**median**	**min**	**max**	**Median**
**La**	215	4511	940	155	5161	904	5.76	63.6	20.6
**Ce**	363	548	456	291	483	417	1.42	11.5	5.46
**Pr**	7.64	9.98	9.06	2.96	9.80	7.20	<LOD	0.15	0.10
**Sm**	120	208	165	226	397	282	<LOD	<LOD	<LOD
**Nd**	1224	1680	1426	148	1985	1398	3.85	9.85	7.68
**Eu**	101	194	146	16.8	47.1	29.7	1.97	9.27	4.71
**Gd**	29.8	97.7	63.2	<LOD	14.8	6.55	<LOD	<LOD	<LOD
**Tb**	29.8	82.4	63.2	<LOD	14.8	6.55	<LOD	<LOD	<LOD
**Dy**	56.9	300	138	13.5	70.2	38.5	<LOD	0.65	0.19
**Ho**	56.6	80.8	69.1	11.2	56.5	26.0	<LOD	5.15	1.59
**Er**	51.9	1214	352	13.3	34.9	22.9	0.18	2.11	1.34
**Tm**	25.6	190	59.0	5.60	11.0	8.13	1.49	6.45	3.14
**Yb**	10.5	179	91.5	10.3	35.0	22.7	1.15	4.35	2.45

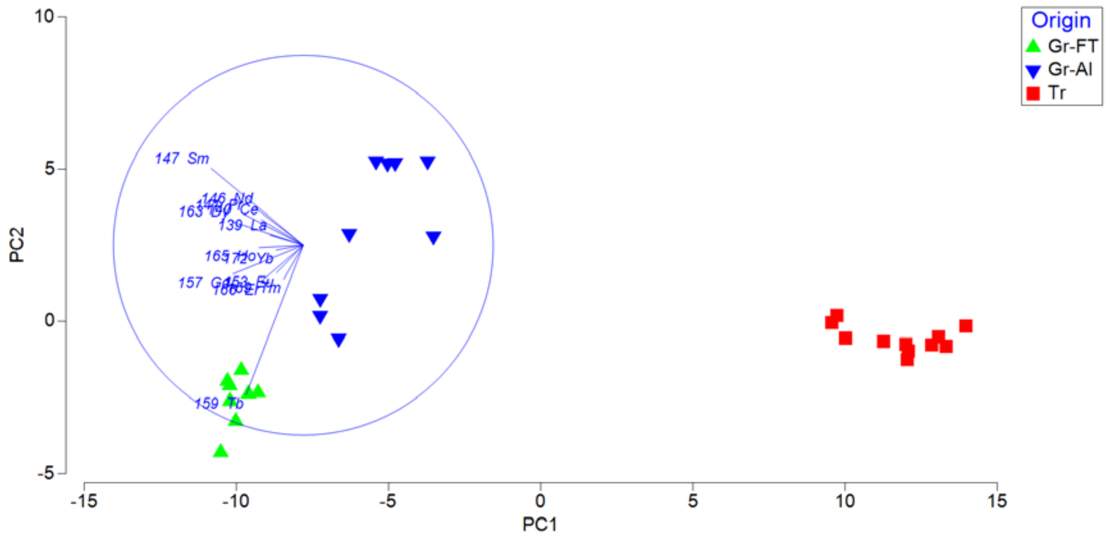

Figure S1. PCA loading plot

Table S2. PCA Results

Principal Component	Eigenvalues	% Variation explained	Cumulative Variation
1	98.1	86.8	86.8
2	7.06	6.2	93.1
3	2.29	2.0	95.1
4	2.08	1.8	97.0
5	1.23	1.1	98.0

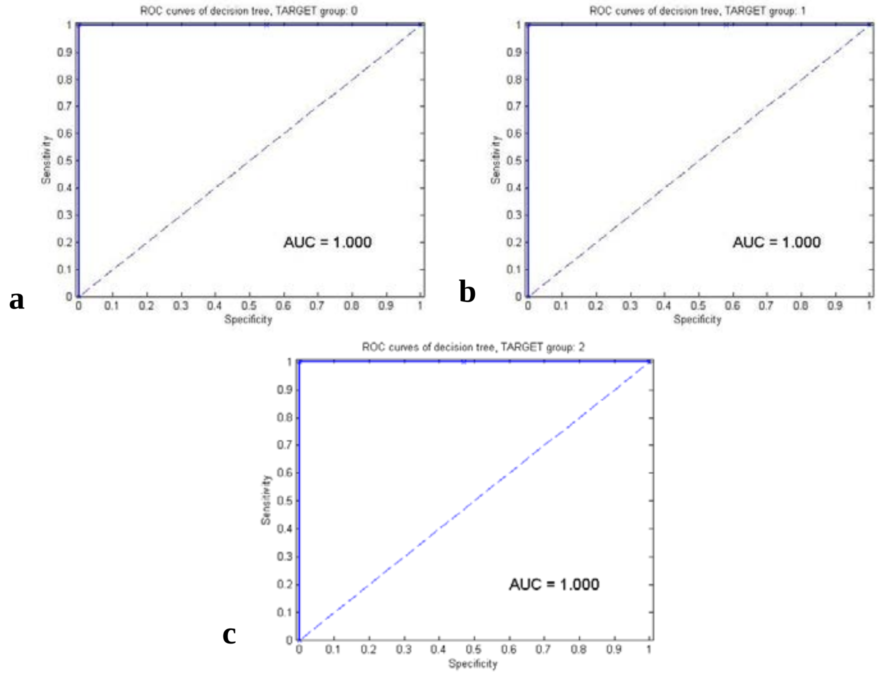

Figure S2. ROC curves for pistachios (a) originating from *Adana*; (b) *Fthiotida*; (c) *Aegina*
